# Prevalence and epidemiology of meningococcal carriage in Southern Ethiopia prior to implementation of MenAfriVac, a conjugate vaccine

**DOI:** 10.1186/s12879-016-1975-3

**Published:** 2016-11-04

**Authors:** Guro K. Bårnes, Paul A. Kristiansen, Demissew Beyene, Bereket Workalemahu, Paulos Fissiha, Behailu Merdekios, Jon Bohlin, Marie-Pierre Préziosi, Abraham Aseffa, Dominique A. Caugant

**Affiliations:** 1 WHO Collaborating Center for Reference and Research on Meningococci, Norwegian Institute of Public Health, Oslo, Norway; 2 Faculty of Medicine, University of Oslo, Oslo, Norway; 3 Armauer Hansen Research Institute, Addis Ababa, Ethiopia; 4 Arba Minch College of Health Sciences, Arba Minch, Ethiopia; 5 Arba Minch General Hospital, Arba Minch, Ethiopia; 6 College of Medicine and Health Sciences, Arba Minch University, Arba Minch, Ethiopia; 7 Department of Methodology Research and Analysis, Norwegian Institute of Public Health, Oslo, Norway; 8 Initiative for Vaccine Research, World Health Organization, Geneva, Switzerland

**Keywords:** *Neisseria meningitidis*, Carriage, *Neisseria lactamica*, Ethiopia, MLST, Meningitis belt, MenAfriVac

## Abstract

**Background:**

*Neisseria meningitidis* colonizes humans and transmits mainly by asymptomatic carriage. We sought to determine the prevalence and epidemiology of meningococcal carriage in Ethiopia prior to the introduction of MenAfriVac, a serogroup A meningococcal conjugate vaccine.

**Methods:**

A cross-sectional meningococcal carriage study was conducted in Arba Minch, southern Ethiopia. A total of 7479 oropharyngeal samples were collected from 1 to 29 year old volunteers, between March and October, 2014. The swabs were cultured for *N. meningitidis* and *Neisseria lactamica* in Ethiopia. *N. meningitidis* isolates were confirmed and characterized by their serogroup, sequence type (ST) and PorA:FetA profile in Norway.

**Results:**

Overall carriage prevalence was 6.6 %. There was no significant difference in overall carriage between male (6.7 %) and female (6.4 %) participants. Highest carriage prevalence (10.9 %) for females was found in the 15–19 years of age, while prevalence among males was highest (11.3 %) in the 20–24 age group. Non-groupable isolates dominated (76.4 %), followed by serogroups X (14.0 %) and W (5.9 %) isolates. No serogroup A was found. Most non-groupable isolates were ST-192. Serogroup W isolates were assigned to the ST-11 clonal complex, and serogroup X isolates to the ST-181 and ST-41/44 clonal complexes. Overall carriage prevalence of *N. lactamica* was 28.1 %. Carriage of *N. meningitidis* and *N. lactamica* varied depending on age and geographic area, but there was no association between carriage of the two species.

**Conclusions:**

Epidemic strains of serogroups W and X were circulating in this area of Ethiopia. As no serogroup A was found among the carriage isolates the immediate impact of mass-vaccination with MenAfriVac on transmission of *N. meningitidis* in this population is expected to be marginal.

## Background

*Neisseria meningitidis*, the meningococcus, is a commensal microorganism colonizing the upper respiratory tract, usually without causing disease. Meningococci are found only in humans and the bacterium is transmitted mainly through close contact and airborne droplets. Carriage prevalence varies geographically, but is usually around 5–10 % [1, 2]. However, in crowded or contained societies, such as universities, carriage prevalence can be significantly higher [3, 4]. Carriage prevalence is usually found to be higher in males than females. In Europe and USA, carriage prevalence is low during childhood and peaks in the adolescence, while carriage is more common among younger children in Africa [5]. Living conditions and social behavior, such as active and passive smoking [6], discotheque visits and crowding [7], are among the known risk factors for carriage.

Occasionally, the bacteria can invade the bloodstream and reach the spinal fluid, and rapidly cause meningitis and/or septicemia [8, 9]. Of 12 known capsular serogroups, serogroups A, B, C, W, X and Y are responsible for nearly all cases of disease. Meningococcal disease occurs endemically throughout the world, but in a region designated as the meningitis belt in sub-Saharan Africa, outbreaks and epidemics occurs every year in the dry season. During these epidemics, carriage prevalence of the outbreak strain can increase by a factor of 10 or higher [10].

Molecular characterization of *N. meningitidis* is performed by multilocus sequence typing (MLST), which assigns isolates to a sequence type (ST) and eventually to a clonal complex, based on the allelic variation in seven housekeeping genes. Classification of variable regions in two outer membrane proteins, PorA and FetA, is also commonly used for additional characterization. In the meningitis belt, serogroup A meningococci of the ST-5 clonal complex have caused most of the epidemics during the past 4 decades [11, 12]. More recently, serogroup X ST-181 and serogroup W ST-11 have also caused large epidemics [13–16], and currently a new serogroup C clone assigned to ST-10217 is spreading in the meningitis belt [17].

*Neisseria lactamica* is a non-pathogenic, closely related species that share the same ecological niche as *N. meningitidis*. Carriers of *N. lactamica* develop cross-reacting systemic opsonophagocytic antibodies to *N. meningitidis* [18] and natural immunity against the meningococci may result from carriage of these closely related commensals*.* Several epidemiological studies have shown an inverse relationship between carriage of *N. lactamica* and meningococci [19–21]. It has been suggested that carriage of *N. lactamica* can inhibit meningococcal carriage [22, 23] and a recent study has shown that inoculation of *N. lactamica* in the oropharynx can both displace current carriage and protect against new acquisition of *N. meningitidis* [22].

As meningococci are transmitted by healthy carriers, conjugated vaccines that prevent colonization, in addition to protecting vaccinated individuals against invasive disease, have shown to be very efficient to control disease [24]. In 2010, a new meningococcal conjugate vaccine, developed specifically for the sub-Saharan meningitis belt, was introduced. The vaccine, MenAfriVac, contains 10 μg serogroup A polysaccharide conjugated to tetanus toxoid, and has been introduced in mass vaccination campaigns of all 1–29 year olds in more than 15 countries. In Ethiopia, one of the countries with high epidemic risk and high disease burden, vaccination has been implemented in 3 phases from 2013 to 2015 [25]. Prior to and in relation to the implementation of the vaccine, several carriage studies have been conducted across the meningitis belt, showing the vaccine’s ability to prevent carriage, and giving useful information about which serogroups and clones that circulate [5, 26–28]. Butajira, in central Ethiopia, was part of one of the carriage studies in the period 2010–2012. Carriage prevalences from 4.3 to 10.0 % were reported [5]. The majority of the isolates were non-groupable (NG), but isolates of serogroup B, C, W, X and Y were also identified [5]. Surveillance of invasive meningitis isolates in Ethiopia in 2012-2013 showed, on the other hand, that in Hawassa, in the southern part of the country, serogroup A was the dominant cause of disease [29].

Therefore, to better understand the epidemiology of meningococcal carriage in southern Ethiopia and to predict the potential impact of MenAfriVac, we conducted a carriage study in advance of the introduction of the vaccine. The results from this carriage study are presented here, together with data on *N. lactamica* carriage, as only a few such studies have been done in sub-Saharan Africa [30–32].

## Methods

### Study design and sampling

**Fig. 1:**
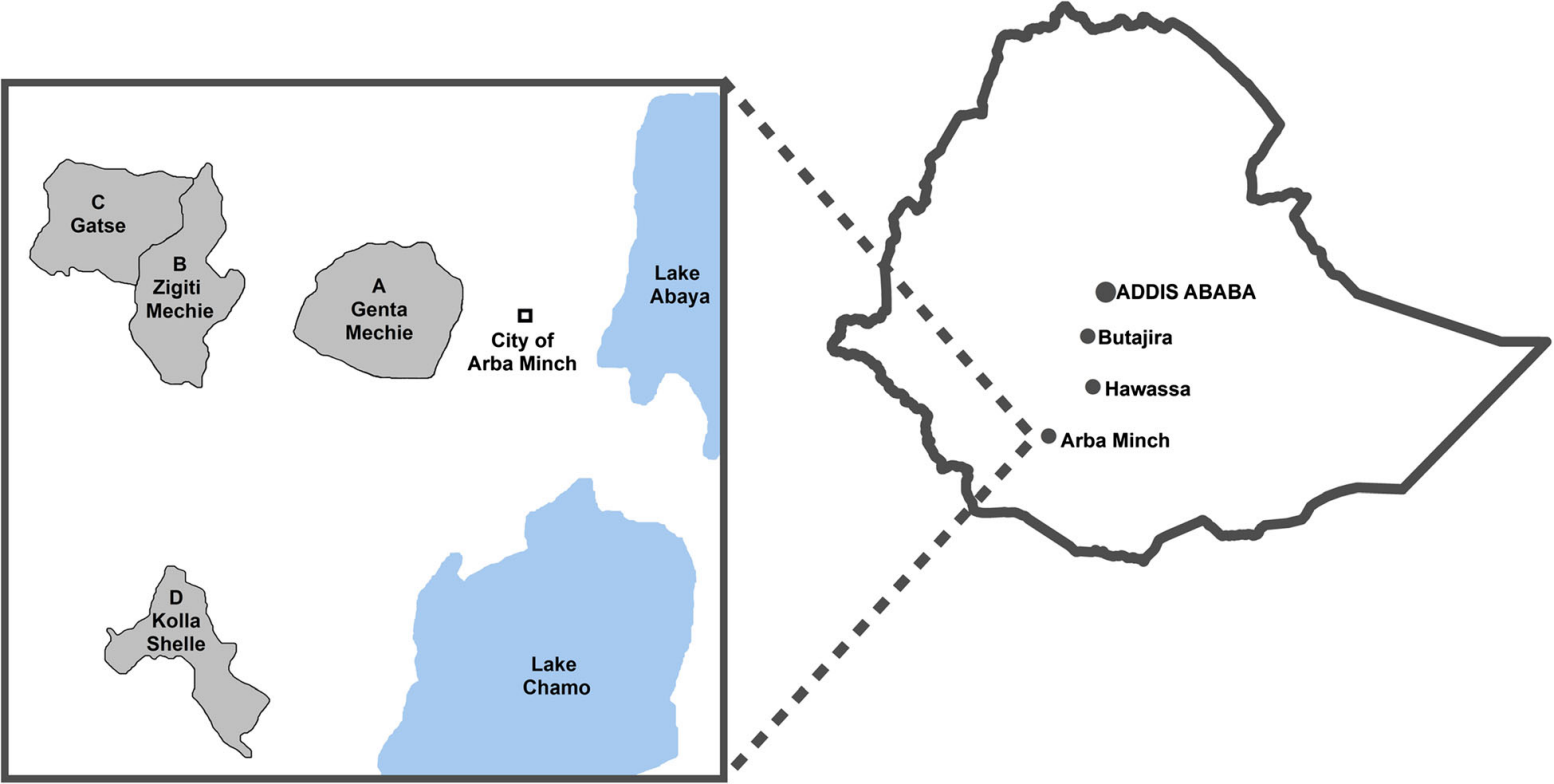
Location of study sites in Ethiopia. (Map created using ArcMAP 10.3.1)

A cross-sectional carriage study was conducted among healthy volunteers aged 1–29 years in the district of Arba Minch in southern Ethiopia during the rainy season, between March 18th and October 1st, 2014. Four kebeles (the smallest administrative unit within a district) were included; Genta Mechie (A), Zigiti Mechie (B), Gatse (C) and Kolla Shelle (D) (Fig. 1). These are all part of the national, community-based Demographic Surveillance System (DSS) in Ethiopia.

Community leaders were consulted and informed about the study prior to sampling. All individuals between 1 and 29 years were invited to participate and recruitment was done by data collectors from the DSS. Demographic data on age and gender were obtained from each participant, together with consent for participation, information regarding meningococcal vaccination in the last 5 years and antibiotic use within the last 30 days.

Sampling took place at open-aired squares or fields centrally located in each village. Oropharyngeal samples were obtained by swabbing the posterior pharyngeal wall and one tonsil with a plain cotton swab (Copan, Italy). Swabs were plated directly onto selective agar plates containing vancomycin, colistin, nystatin, trimethoprim lactate (VCNT, Becton, Dickinson, NJ, US) and Vitox supplement (Thermo Fisher Scientific, MA, US) in the field, before being transported in CO_2_-enriched containers, at ambient temperature, to the microbiology laboratory at Arba Minch General Hospital within 6 h, for incubation at 37 °C overnight.

### Laboratory analysis

Identification of *N. meningitidis* and *N. lactamica* was made by examination of colony morphology on the VCNT plates, subculture on blood agar plates, oxidase reaction, Gram staining and enzymatic testing for β-galactosidase (ONPG) and γ-glutamyltransferase (GGT) [33]. *N. meningitidis* isolates were serogrouped by conventional slide agglutination (Remel, GA, USA). The meningococcal strains were stored in Greaves solution [34] at −80 °C, and transported on dry ice for confirmatory re-analysis and further characterization using molecular methods at the Norwegian Institute of Public Health (NIPH).

According to a previously established quality control scheme [35], randomly chosen non-meningococcal isolates were used as external quality control samples. These were collected during the identification process, both at the stage of colony morphology and at the stage of enzymatic testing, and were re-analyzed at NIPH. The final results on meningococcal carriage, including molecular characterization, were based on confirmed results from NIPH only.

### Molecular characterization

DNA from the isolates was extracted by suspension in Tris-ethylenediaminetetraacetic acid (EDTA) buffer (10 mM Tris-HCl and 1 mM EDTA), pH 8.0, heating at 95 °C for 10 min and centrifugation at 16,000 × g for 5 min. DNA from the supernatant was amplified by PCR, and MLST was performed according to the method on the website (http://pubmlst.org/neisseria/) [36]. Classification by outer membrane protein PorA and FetA variants was done by sequencing of the *porA* [37] and *fetA* [38] genes. New MLST alleles, STs, PorA and FetA variants were submitted to the MLST database. PCR analysis of capsule coding genes was undertaken for serogroup determination of isolates with questionable slide agglutination results. Isolates assigned to new STs and a subset of isolates assigned to the ST-53 and ST-192 clonal complexes, known to harbor the *cnl* locus [39], were also included in capsule PCR analysis.

### Phylogeny

The concatenated sequences from the MLST analysis were aligned with MAFFT [40] and non-informative sites were removed prior to phylogenetic analyses. Maximum likelihood (ML) estimation was subsequently performed to estimate the optimal nucleotide substitution matrix by the PhyML program [41] within the R-package “Ape” [42]. The generalized time reversible (GTR) substitution matrix was found to be optimal as assessed with the Akaike information criterion and used with the RAxML program [43] to create a ML-tree. The tree was bootstrapped 500 times.

### Data management and statistical analyses

Demographic data from participants and laboratory results were merged in a Microsoft Access database at the NIPH. Samples without complete traceability were excluded from the analysis.

Data examination and creation of tables and graphs were performed using Microsoft Excel 2010. Comparison of prevalence between groups was done with a chi-square test in IBM SPSS Statistics 23 statistical software. Remaining statistical analyses were performed using the statistical programming language R [44]. A *p*-value <0.05 was considered statistically significant.

To study factors affecting carriage, a proportional binomial generalized additive regression model (GAM) [45] was fitted with the number of *N. meningitidis* positives and negatives for each age *i = 1–29* as the response. Sex (male/female) and kebele (A, B, C and D) were added as categorical covariates and age was fitted with a spline function *f()*:

$$ {y}_i\sim Bin\left({n}_i,\ {\pi}_i\right) $$

$$ logit\left({\pi}_i\right)\sim {\beta}_0+f\left(Ag{e}_i\right) + {\beta}_1Se{x}_i + {\beta}_2 Kebel{e}_i $$

where *y*_*i*_ is the number of positives for each age *i*, *π*_*i*_ is the probability of being infected and *n*_*i*_ is the number of samples. Goodness of fit was assessed by *R* ^*2*^ (GAM) and using the Akaike information criterion.

Simpson’s Index of Diversity was calculated based on serogroup, ST-complex, STs, PorA and FetA variants to determine the diversity of the meningococcal isolates by kebele. The index ranges from 0 to 1, with higher values indicating greater diversity, and was calculated as described previously [46].

## Results

### Study population and samples

A total of 7722 individuals from 4 different kebeles was enrolled in the study and complete linked participant information was obtained for 7479 (96.8 %), who were then included in the analyses (Table 1). There was an equal participation of males (*n* = 3740) and females (*n* = 3739). Age ranged from 1 to 29 years and the majority of the participants, 58.1 %, were below 10 years of age. The average and median ages were 11.0 and 9.0 years for females, slightly older than for males, which were 9.3 and 8.0 years, respectively.

**Table 1:** Demographic data of study participants, in percent

			Kebele^a^			
		Total	A	B	C	D
		(*n* = 7479)	(*n* = 1307)	(*n* = 2479)	(*n* = 2161)	(*n* = 1532)
Gender	Male	50.0	50.7	51.3	50.1	47.3
	Female	50.0	49.3	48.7	49.9	52.7
Age	1–4	21.1	16.7	22.7	23.3	19.0
	5–9	37.0	33.4	40.9	35.1	36.4
	10–14	22.4	26.4	23.5	19.8	20.9
	15–19	6.6	9.3	5.6	4.3	8.9
	20–24	4.4	4.6	3.0	4.1	7.0
	25–29	8.6	9.6	4.4	13.4	7.8

^a^Kebele: Genta Mechie (A), Zigiti Mechie (B), Gatse (C) and Kolla Shelle (D)

None of participants in kebeles A, B and C reported to have been vaccinated with any meningococcal vaccine in the last 5 years, whereas 5.6 % of the participants (*n* = 86) in kebele D had been vaccinated with a meningococcal A + C polysaccharide vaccine in a reactive mass vaccination campaign by Ethiopian health authorities following an epidemic in 2013. Except for 2 participants in kebele D, no others reported the use of antibiotics in the last 30 days prior to sampling.

### Laboratory quality control

To ensure correct estimation of carriage prevalence, 5 % of all primary samples assessed to be non-meningococcal at the laboratory in Arba Minch were stored for external quality control. These samples were re-analyzed at NIPH and none were identified as meningococci. Of 591 isolates identified as *N. meningitidis* in Arba Minch, 492 isolates were confirmed as such at NIPH (83.2 %). The final results on meningococcal carriage were based on confirmed results from NIPH, leaving no false negative or false positive meningococcal samples in the data analyses.

### Meningococcal carriage epidemiology

**Fig. 2:**
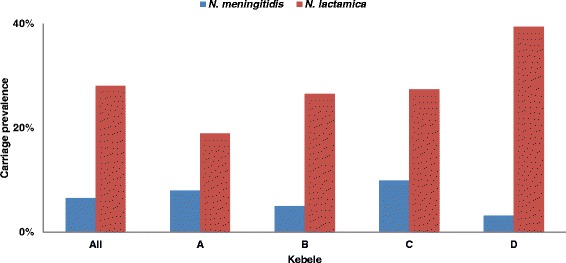
Carriage prevalence of *N. meningitidis* and *N. lactamica*, in total and by kebele, in Arba Minch, Southern Ethiopia

**Fig. 3:**
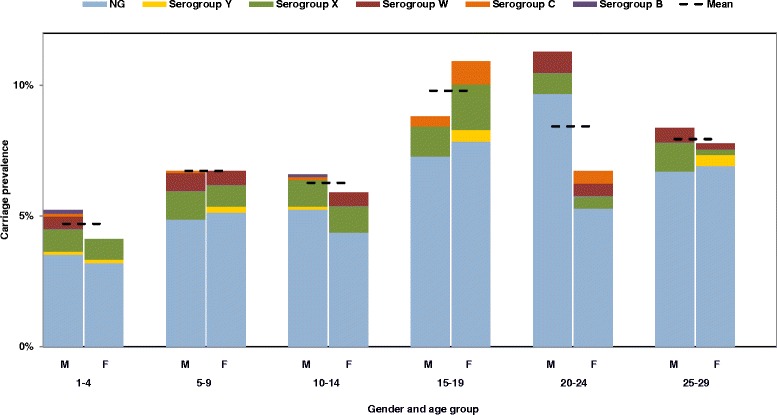
Meningococcal carriage prevalence by gender, age and serogroup in Arba Minch, Southern Ethiopia. (NG, non-groupable)

**Fig. 4:**
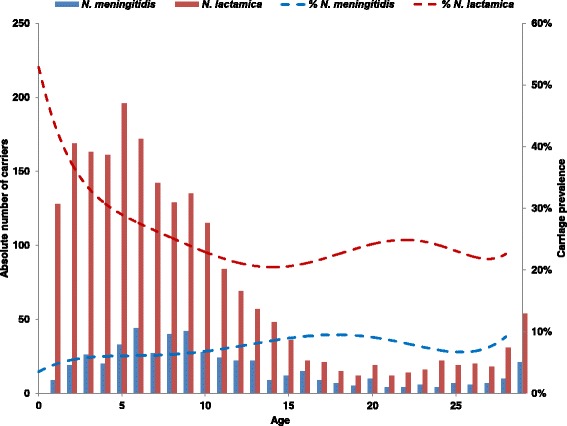
Absolute (bars) and percent (*dotted trend line*) carriage of *N. meningitidis* and *N. lactamica* carriage by age in Arba Minch, Southern Ethiopia

Overall carriage prevalence was 6.6 %. Prevalence ranged from 3.2 % in kebele D to 9.9 % in kebele C, varying significantly between the different kebeles (Fig. 2, Table 2). There was no overall significant difference in carriage between male (6.7 %) and female (6.4 %) participants (Fig. 3, Table 2). Carriage incidence varied by age (Fig. 4, Table 2) and the overall peak prevalence was seen in the 16 year olds. For females, the highest carriage prevalence (10.9 %) was found in the 15–19 years of age, while among males, prevalence was highest (11.3 %) in the 20–24 age group (Fig. 4).

**Table 2:** Factors affecting carriage of *Neisseria meningitidis* and *Neisseria lactamica* in Arba Minch, Southern Ethiopia

		*Neisseria meningitidis*			*Neisseria lactamica*		
Covariate		Estimate	95 % CI	*P*	Estimate	95 % CI	*P*
Sex^a^		0.06	(−0.13, 0.25)	0.536	0.04	(−0.06, 0.15)	0.439
Kebele^b^	A						
	B	−0.41	(−0.68, −0.14)	0.001	0.37	(0.20, 0.53)	<0.001
	C	0.28	(0.03, 0.52)	0.028	0.42	(0.25, 0.60)	<0.001
	D	−0.95	(−0.60, −1.30)	<0.001	1.01	(0.84, 1.19)	<0.001
*Smooth term age*		edf^c^	*X* ^2^	*P*	edf	*X* ^2^	*P*
f(Age)		2.31	14.78	0.002	3.54	178	<0.001
*Goodness of fit*		R^2^	Deviance explained		R^2^	Deviance explained	
		0.27	29.2 %		0.64	59.6 %	

^a^Male used as reference

^b^Kebele A used as reference

^c^Estimated degrees of freedom

Carriage prevalence among individuals from kebele D who were vaccinated with a meningococcal A + C polysaccharide vaccine in 2013 was higher (5.8 %, *n* = 5/86) than among those who were not vaccinated (3.0 %, *n* = 44/1446), but the difference was not statistically significant, confirming that polysaccharide vaccines have no long term, if any, impact on carriage [47]. Among those previously vaccinated, 2 were carriers of serogroup C and 3 of NG meningococci.

We identified a total of 376 NG isolates, which represented 76.4 % of all isolates, and a carriage prevalence of NG meningococci of 5.0 %. Among the encapsulated isolates, serogroup X dominated with a prevalence of 0.9 %, followed by serogroups W, Y, C and B (Table 3). None of the isolates were assigned to serogroup A.

**Table 3:** Carriage of *Neisseria meningitidis* among 7479 individuals, 1–29 year old, in 4 kebeles in Arba Minch, Southern Ethiopia

			Kebele^a^							
	Total		A		B		C		D	
	*n*	%	*n*	%	*n*	%	*n*	%	*n*	%
Total	492	6.58	104	7.96	125	5.04	214	9.90	49	3.20
Serogroup B	2	0.03	0	0.00	0	0.00	1	0.05	1	0.07
Serogroup C	7	0.09	1	0.08	0	0.00	2	0.09	4	0.26
Serogroup W	29	0.39	12	0.92	8	0.32	7	0.32	2	0.13
Serogroup X	69	0.92	2	0.15	14	0.56	35	1.62	18	1.17
Serogroup Y	9	0.12	3	0.23	3	0.12	2	0.09	1	0.07
Non-groupable	376	5.03	86	6.58	100	4.03	167	7.73	23	1.50

^a^Kebele: Genta Mechie (A), Zigiti Mechie (B), Gatse (C) and Kolla Shelle (D)

**Fig. 5:**
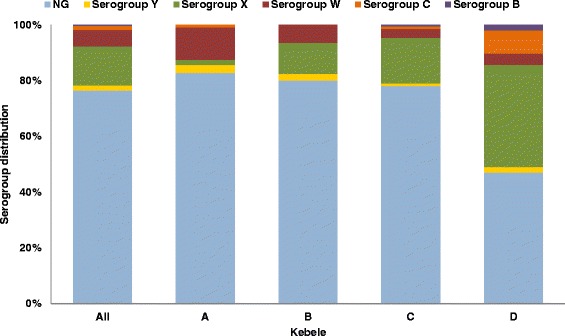
Distribution of meningococcal serogroups by kebele in Arba Minch, Southern Ethiopia. (NG, non-groupable)

The serogroup distribution varied between the 4 kebeles (Fig. 5). Serogroup W, X, Y and NG isolates were found in all kebeles, whereas the less frequent serogroups, serogroup C and serogroup B were found only in kebeles A, C and D, or kebeles C and D, respectively. Kebeles A, B and C, which are connected to each other and to Arba Minch city by the same road (Fig. 1), had the highest proportions of NG isolates (Fig. 5). On this same geographical axis, the proportion of W isolates declined with increasing distance from the city, from 11.5 % in kebele A, to 6.4 % and 3.3 % in kebeles B and C, respectively. The proportion of serogroup X isolates, on the other hand, increased with the distance from the city: 1.9 %, 11.2 % and 16.8 % in kebeles A, B and C, respectively. In kebele D, the distribution was different; the proportion of NG isolates were lower (46.9 %) and there was a higher proportion of serogroup X and C isolates (36.7 % and 8.2 %, respectively).

### Molecular characterization

The strain collection was composed of a total of 32 STs; 25 STs belonging to 11 different clonal complexes, and 7 STs unassigned to a clonal complex (Table 4). Among the 492 meningococcal isolates, we identified 8 new alleles, 13 new STs, 4 new PorA variants and 4 new FetA variants. All new STs and alleles were submitted to the pubMLST website [48].

**Table 4:** Molecular characteristics of meningococcal carriage isolates in 4 kebeles in Arba Minch, Southern Ethiopia

Sero-group	ST-complex	ST	PorA	FetA	n	Kebele^a^			
						A	B	C	D
B	35	35	P1.22-1,14	F4-1	1				1
		10450	P1.22-1,14	F4-1	1			1	
C	103	11592	P1.5-1,10-4	F3-9	5			2	3
	35	35	P1.22-1,14	F4-1	1				1
	175	8447	P1.5-1,2-2	F5-8	1	1			
W	11	11	P1.5,2	F1-1	26	12	7	5	2
				F1-7	1		1		
		2724	P1.5,2	F1-1	1	1			
		7849	P1.5,2	F1-1	1	1			
X	181	181	P1.5-1,10-1	F3-22	4			4	
			P1.5-1,10-1	F1-7	2			2	
			P1.5-1,10-1	F4-23	1		1		
		11372	P1.5-1,10-1	F4-23	48	2	12	21	13
		11591	P1.5-1,10-1	F4-23	6			1	5
	41/44	207	P1.12-1,13-2	F5-169	3		1	2	
				F1-7	2			2	
				F1-21	1			1	
				F3-60	1			1	
		1053	P1.12-1,13-2	F5-169	1			1	
Y	167	2880	P1.5-1,10-1	F1-3	8	3	3	1	1
		767	P1.5-1,10-1	F1-3	1			1	
NG	167	2880	P1.5-1,10-8	F1-3	9	1	2	5	1
	175	175	P1.22-11,15-25	F5-1	1		1		
			P1.22-11,15-56	F5-1	3			3	
		8447	P1.5-1,2-2	F5-8	1			1	
	178	178	P1.19,15	F5-8	1				1
	181	11372	P1.5-1,10-1	F4-23	1				1
	192	192	P1.18-11,42-1	Neg	284	79	77	118	10
		11598	P1.18-11,42-1	Neg	1			1	
		11642	P1.18-11,42-1	Neg	1			1	
	198	198	P1.18,25-11	F5-5	3		2		1
			P1.25-11,38-1	F5-5	1		1		
		11741	P1.18,25-37	F1-7	1				1
			P1.18,25-65	F1-7	2		1		1
	35	35	P1.22-1,14	F4-1	4			1	3
	53	53	P1.7,3	F1-7	2			2	
			P1.7,30	F1-3	2			2	
				F1-49	1			1	
				F1-7	7		2	5	
				F3-69	1		1		
			P1.7,30-2	F1-215	1			1	
				F1-7	4		1	3	
				F2-28	1		1		
				F5-9	1			1	
			P1.7,30-3	F1-31	1		1		
				F1-7	5			5	
			P1.7,30-4	F1-7	1			1	
			P1.7,30-4	F1-7	1			1	
			P1.7-11,30	F1-21	2		2		
				F3-69	1			1	
				F6-6	1			1	
			P1.7-11,30-2	F1-21	1		1		
			P1.7-11,30-3	F1-3	1			1	
			P1.7-2,30	F1-182	2		2		
			P1.7-2,30-3	F1-7	2		2		
			P1.7-2,30-6	F1-7	1		1		
		2075	P1.7,30	F1-113	1			1	
			P1.7-2,30	F1-153	2	2			
				F3-28	1		1		
			P1.7-2,30-3	F1-153	3	3			
			P1.7-2,30-5	F1-153	1	1			
			P1.7-54,30	F1-24	1			1	
		7389	P1.7-2,30	F1-7	1		1		
		11594	P1.7,30-2	F1-7	1			1	
	UA^b^	11587	P1.18-1,3-8	F4-72	1				1
			P1.18-11,42-1	F5-102	1			1	
		11595	P1.18-1,3	F4-72	4			4	
		11597	P1.22-11,15-25	F1-36	2			2	
		11624	P1.18-1,3	F4-72	1			1	
		11638	P1.18-1,3-8	F4-72	1			1	
	UA	11593	P1.22-1,14	F5-19	1				1
		11617	P1.22-11,15-57	F5-7	2				2

*ST* sequence type, *CC* clonal complex, *n* number of isolates, *NG* non-groupable, *UA* unassigned to any clonal complex, *Neg* negative

^a^Kebele: Genta Mechie (A), Zigiti Mechie (B), Gatse (C) and Kolla Shelle (D)

^b^UA cluster related to ST-11587

The majority of isolates (57.8 %, *n* = 284) was assigned to ST-192, with PorA variant P1.18-11,42-1, and lacking FetA. All of these isolates were NG (Table 4) and, thus, carriage prevalence of this specific clone was 3.8 %. Two isolates were single-locus variants of ST-192 and were, together with a subset of 147 ST-192 isolates (52 %), subjected to PCR of the capsule locus; all of them harbored the *cnl* locus. All 50 NG isolates belonging to the ST-53 clonal complex were also capsule null (Table 4), as was a cluster of NG isolates closely related to ST-11587.

The majority of serogroup X isolates were assigned to the ST-181 clonal complex, being either ST-181 (*n* = 7), ST-11372 (*n* = 48) or ST-11591 (*n* = 6) (Table 4). Seven serogroup X isolates were assigned to ST-207 of the ST-41/44 clonal complex; all had the same PorA variant, but 4 different FetA variants were identified.

All 29 serogroup W isolates were assigned to ST-11 clonal complex (Table 4) and most of these (*n* = 27) were assigned to the hypervirulent ST-11 clone, known to cause large epidemics [49].

Of the 7 serogroup C isolates, five were assigned to the ST-11592, a new ST of the ST-103 complex (Table 4). Only 2 serogroup B isolates were identified, both assigned to the ST-35 clonal complex. The 9 serogroup Y isolates belonged to the ST-167 complex.

**Fig. 6:**
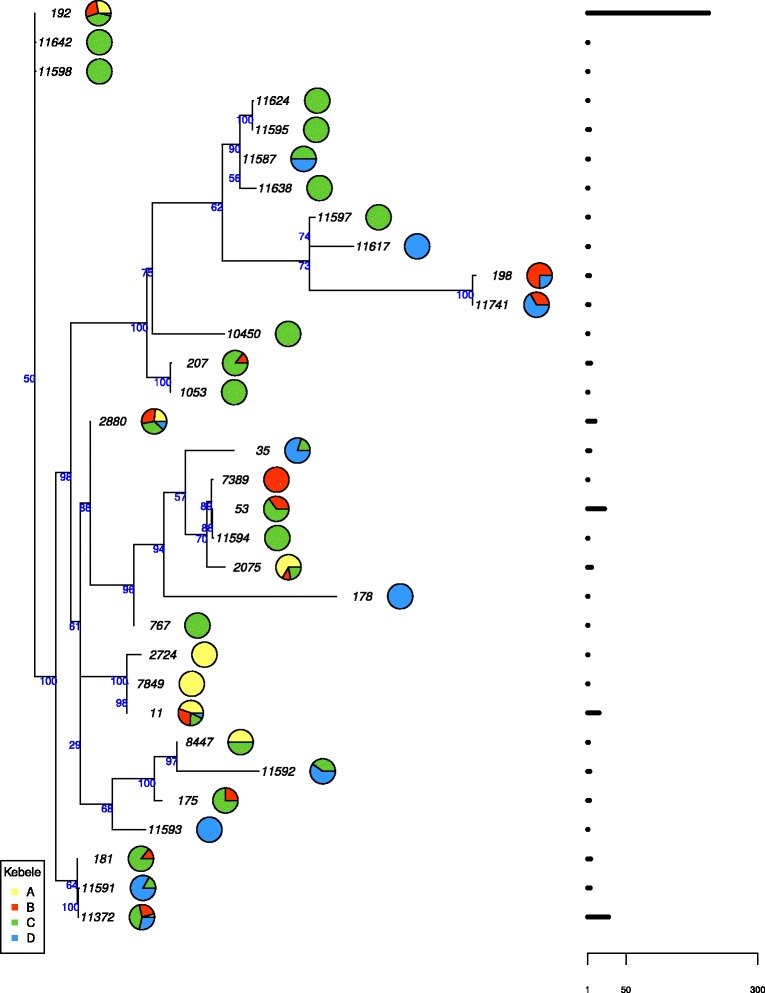
Phylogenetic tree of meningococcal sequence types (STs) in Arba Minch, Southern Ethiopia. Pie charts indicate the distribution by kebele and the horizontal bars on the right represent the total number of isolates. The node numbers designate the percentage of bootstrap replications

The most frequent STs were found in all kebeles, except ST-53 which was found in kebele B and C only. All STs represented by 5 or more isolates were present in more than one kebele. The majority of the 16 STs found only in one kebele were new STs (*n* = 13) and were found mostly in kebele C (*n* = 10). Isolates from kebele C are spread across the phylogenetic tree, while isolates from the other kebeles are more clustered in the centre and lower part of the tree (Fig. 6). The diversity of meningococcal isolates varied between kebeles and kebele C had the highest number of STs (*n* = 24) and genotypes, defined as unique ST and PorA/FetA combinations (*n* = 41) (Fig. 6, Table 4). Simpson’s Index of Diversity, ranging from least to most diverse, was estimated to 0.433 for kebele A, 0.610 for kebele B, 0.679 for kebele C and 0.880 for kebele D.

### Carriage of *N. lactamica*

Results from morphological and enzymatic testing performed in Arba Minch were used to estimate the carriage prevalence of *N. lactamica* (oxidase-positive, ONPG-positive and GGT-negative isolates). Overall prevalence was 28.1 %, with significant differences between the 4 kebeles (Table 2), ranging from 18.9 % in kebele A to 39.4 % in kebele D (Fig. 2). *N. lactamica* carriage varied with age (Fig. 4, Table 2). The highest prevalence was seen in 1 year old children (54.5 %) and declined until the age of 16 years (20.2 %), when carriage increased again for females while staying low for males. However, there was no statistical difference in overall *N. lactamica* carriage between females (27.6 %) and males (28.5 %) (Table 2). The ratio between carriage of *N. lactamica* and *N. meningitidis* varied from 0.42 in kebele A to 0.08 in kebele D and there was no statistically significant association between carriage of *N. lactamica* and *N. meningitidis*. There was no difference in carriage prevalence of *N. lactamica* between those who had been vaccinated with a meningococcal A + C vaccine and those who had not in kebele D (*p* = 0.61).

## Discussion

We present here the epidemiology and molecular characterization of meningococcal carriage isolates retrieved from 7479 children and young adults living in southern Ethiopia, immediately before the introduction of MenAfriVac. Carriage prevalence was 6.6 % and NG meningococcal isolates dominated. No serogroup A isolates were detected, but clones of other encapsulated meningococci with epidemic potential circulated: serogroup W ST-11 and serogroup X ST-181.

Despite the study being performed in the southern part of Ethiopia where serogroup A disease had been reported recently [29], no carriage of serogroup A was found. This absence of serogroup A meningococci in Ethiopia, as well as the low carriage prevalence of serogroup A meningococci in several other countries across the meningitis belt, was also shown in another recent study [5]. The introduction of the monovalent serogroup A conjugate vaccine is thus expected to have very limited measurable impact on transmission of the pathogen and prevalence of meningococcal disease. Therefore, the implementation of MenAfriVac in Ethiopia might not have an immediate public health impact, as strains with epidemic potential of other serogroups are currently circulating in the population.

The meningococcal carriage prevalence was comparable to that found in other studies in the region [2, 5, 50]. Overall, the serogroup distribution was consistent with results obtained by others during the rainy season in Butajira, situated north of Arba Minch [5], although there was a lower proportion of carriers with serogroup Y and a higher proportion of carriers with serogroup X in our study. The circulation of meningococcal strains with the potential to cause large outbreak, such as ST-11 and ST-181, is of concern, and the data from this study contribute to inform the Ethiopian health authorities of what can be expected in case of increasing incidence of meningococcal disease. Serogroup W ST-11 is the dominating pathogenic clone in most of the meningitis belt countries after serogroup A disease was practically eliminated following mass vaccination with MenAfriVac. Serogroup X ST-181 has also shown potential for causing large outbreaks and to date there is no available vaccine protecting against this serogroup. One of the new STs identified in this study, ST-11592 (serogroup C) was also identified among patients in East-Ethiopia in November 2015 (unpublished data). This serogroup C genotype is not related to the epidemic clone ST-10217 currently spreading serogroup C disease in Nigeria and Niger [17] and its epidemic potential is unknown.

The high carriage prevalence of non-groupable meningococci proves that the bacterial capsule is not a requirement for person-to-person transmission. Although large epidemics are caused by encapsulated bacteria, non-groupable meningococci are also capable of causing invasive disease [51–53].

The study shows that there are geographic variations in both prevalence and serogroup distribution, even within a small area. Kebele D differed most from the other three sites with a significantly lower prevalence of *N. meningitidis* and a higher proportion of encapsulated bacteria. Kebele D is situated in the low land and is more urbanized than the other three sites. The other study sites are all in the highlands, with different temperature and climatic conditions, and are situated along the same road starting in Arba Minch city. Interestingly, the prevalence of serogroup W decreased with the distance from the city, suggesting that this serogroup was spreading from there, while the opposite was found for serogroup X. The most frequent STs were found in all study areas, and with one exception (ST-11372), all the new STs discovered in this study were found only in one or two kebeles. The majority of the new STs were found in kebele C, the kebele which is situated furthest away from the city of Arba Minch. This was also the kebele with the highest carriage prevalence and the highest diversity of meningococcal isolates. These observations indicate that new STs emerge locally and, in the absence of large epidemics, meningococcal carriage epidemiology shows greater local variation.

Despite identifying more than 30 different STs among the carriage strains, the diversity of the strain collection was relatively low, as 85 % of the isolates were assigned to one of 4 STs, and 97 % belonged to one of 19 STs. However, the diversity was higher than reported from Burkina Faso [50] and this is in line with what have previously been observed, that the diversity is higher in countries on the edges of the meningitis belt, as compared to countries located centrally in the belt [5].

The carriage prevalence of *N. lactamica* was comparable to that found in a study in Burkina Faso using the same methodology [30], but significantly higher than in a recent study in Ethiopia and other African countries, which defined *N. lactamica* based on ribosomal protein L6 gene (*rplF*) sequencing [32]. The prevalence of *N. lactamica* carriage reported in older studies varied greatly between settings [20, 21, 31, 54], as well as between geographically nearby areas [55]. The overall age distribution was consistent with previous studies, except that we found the highest prevalence in the 1 year olds, whereas most others report an increasing carriage rate through the first 2 years of life [19, 30, 32, 55, 56]. Contrary to what have been suggested by others, [19–21, 32], we found no association between the proportion of carriage of *N. lactamica* and *N. meningitidis*. Additionally, the ratio of carriage prevalence between the two species varied greatly from kebele to kebele; high *N. lactamica* carriage prevalence did not imply low *N. meningitidis* carriage or vice-versa.

Oropharyngeal swabbing combined with either direct plating, as used in this study, or short transportation time of the swab, improves detection of meningococcal carriage [57]. No false negative isolates were identified among the presumed non-meningococcal external quality control samples. We therefore believe the sampling and sample treatment were good in this study, despite difficult field conditions. The true carriage prevalence in the population is, however, likely to be higher than reported, as sensitivity of swabbing and culture is estimated to be 60–83 % [58]. The use of PCR for direct detection of meningococci from oropharyngeal swabs, in addition to culturing, may have increased the number of detected carriers [59].

## Conclusions

No *N. meningitidis* belonging to serogroup A was detected among 7479 healthy carriers in the Arba Minch area in Ethiopia in 2014. Thus, the immediate public health impact of the introduction of the monovalent serogroup A conjugate vaccine later the same year is not expected to be measurable as it was in other countries, where serogroup A circulation was stopped and herd protection established [26, 27].

*N. meningitidis* belonging to ST-192 with neither capsule nor FetA expression were most frequently found, showing that these surface-exposed components are not required for successful transmission.

Carriage of *N. meningitidis* and *N. lactamica* was influenced by age and geography, even within a small geographic area*.*

The presence of epidemic strains assigned to the virulent clonal complexes ST-11 (serogroup W) and ST-181 (serogroup X) highlights the need for multivalent conjugate vaccines covering these serogroups.

## Abbreviations

AHRI: Armauer Hansen Research Institute
ALERT: All-Africa Leprosy, Tuberculosis and Rehabilitation Training Centre
DSS: Demographic Surveillance System
EDTA: Ethylenediaminetetraacetic acid
GAM: Generalized additive regression model
GGT: γ-glutamyltransferase
GTR: generalized time reversible
ML: Maximum likelihood
MLST: Multilocus sequence typing
NG: Non-groupable
NIPH: Norwegian Institute of Public Health
ONPG: β-galactosidase
PCR: Polymerase chain reaction
ST: Sequence type
VCNT: Medium containing vancomycin, colistin, nystatin and trimethoprim lactate
